# Brolucizumab in recalcitrant neovascular age-related macular degeneration–real-world data in Chinese population

**DOI:** 10.1371/journal.pone.0301096

**Published:** 2024-04-02

**Authors:** Chang-Chi Weng, Sheng-Chu Chi, Tai-Chi Lin, Yi-Ming Huang, Yu-Bai Chou, De-Kuang Hwang, Shih-Jen Chen

**Affiliations:** 1 Department of Ophthalmology, Taipei Veterans General Hospital, Taipei City, Taiwan; 2 School of Medicine, National Yang Ming Chiao Tung University, Taipei City, Taiwan; National Taiwan University, TAIWAN

## Abstract

This retrospective study aimed to determine the short-term efficacy and safety of brolucizumab treatment for recalcitrant neovascular age-related macular degeneration (nAMD) in a real-world setting in Taiwan. Recalcitrant nAMD patients who were treated with brolucizumab from November 2021 to August 2022 at Taipei Veterans General Hospital were included. Patients were followed for 3 months after switching to brolucizumab. The primary outcomes were changes in mean best-corrected visual acuity (BCVA) and central retinal thickness (CRT) from baseline to the third month. The secondary outcomes included the incidence of intraocular inflammation (IOI), proportion of patients with subretinal and intraretinal fluid (SRF and IRF), and change in pigment epithelial detachment (PED) height from baseline to the third month. The significance level was considered as p < .05 in all tests. A total of 38 patients (40 eyes) with a mean (±SD) age of 76.3 (±10.84) years were included. The baseline BCVA was 0.92±0.64 logMAR, and the CRT and PED height were 329.0±171.18 and 189.8±114.94 um, respectively. The patients had a significant reduction in CRT and resolution of IRF and SRF from baseline to the third month. There were numerical improvements in mean BCVA and PED height, but they were not significant. The percentages of achieving at least 0.1, 0.2, and 0.3 logMAR (equivalent to 5, 10, 15 ETDRS letters) visual gain were 50%, 37.5%, and 30%, respectively, during the first 3 months of follow-up. No IOI occurred in these patients. This study demonstrated that brolucizumab had good short-term structural and functional efficacy in recalcitrant nAMD patients.

## Introduction

Age-related macular degeneration (AMD) is a leading cause of blindness worldwide [1, 2]. Neovascular age-related macular degeneration (nAMD) is the advanced stage of AMD, and it causes severe vision loss [2]. Anti-vascular endothelial growth factor (VEGF) therapy is now the standard of care for patients with nAMD, and it has improved patient outcomes effectively ever since its introduction [3, 4].

Nevertheless, there are still unmet needs with current anti-VEGF therapy. For instance, frequent injections of anti-VEGF and clinic visits increase the burden for both patients and physicians, which has led to undertreatment in real-world settings [5]. In addition, an incomplete response to anti-VEGF therapy and persistent fluid in 20–50% of patients despite monthly anti-VEGF injections are also causes of suboptimal vision gain [6].

The HAWK and HARRIER phase 3 trials of brolucizumab for nAMD demonstrated non-inferior visual outcomes, longer duration, and greater reductions in central retinal thickness (CRT) and retinal fluid compared to aflibercept [7, 8]. In addition, even though brolucizumab has been reported to be associated with a higher rate of intraocular inflammation (IOI), it can still relieve the burden of frequent injections after proper patient education and monitoring for IOI [8, 9]. Considering its potent efficacy in reducing retinal fluid, some short-term real-world studies focusing on the response of recalcitrant nAMD have shown significant fluid reduction but controversial visual outcomes [10–15]. However, there are still few real-world results of the efficacy and safety profile of brolucizumab in Chinese populations.

This retrospective study provides the real-world Taiwanese data of brolucizumab treatment in patients with nAMD refractory to other anti-VEGF agents. The objectives were to explore the short-term efficacy and safety outcomes of brolucizumab in patients with recalcitrant nAMD in Taiwan and expand the applicability of current real-world data.

## Material and methods

This retrospective study was approved by the Institutional Review Board of Taipei Veterans General Hospital (TPEVGH IRB number: 202212014CC) on January 18^th^ 2023 and was conducted in accordance with the Declaration of Helsinki. The Institutional Review Board of Taipei Veterans General Hospital approved the waiving of patient informed consent due to the retrospective nature of the review. Patient data was accessed for research purposes from January 19^th^ 2023 and maintained confidentially. All authors had access to subject information during data collection. Recalcitrant nAMD patients who were treated with 6mg brolucizumab in the operating room from November 2021 to August 2022 at Taipei Veterans General Hospital were included. Patients who were followed for 3 months after receiving brolucizumab therapy were included, and the first 3 months of data were analyzed. Recalcitrant nAMD was defined as patients who had nAMD with persistent intraretinal or subretinal fluid (IRF or SRF) despite a high frequency of intravitreal injections of other anti-VEGF agents (bevacizumab, ranibizumab, or aflibercept) over a longer period of time prior to the switch to brolucizumab [12]. The patients had received at least three injections of other anti-VEGF agents, with the last injection administered within two months before switching to brolucizumab. Patients with polypoidal choroidal vasculopathy (PCV) confirmed by indocyanine green angiography who had a poor response after frequent injections of anti-VEGF were also included. The exclusion criteria included eyes with coexisting vitreoretinal pathology other than nAMD/PCV, choroidal neovascularization due to any other etiology, significant media opacities that precluded observation of the ocular fundus, and inability to undergo a visual acuity test.

All of the included patients underwent comprehensive ophthalmic examinations, including best-corrected visual acuity (BCVA) measured at a distance of 6 meter away from a Snellen chart and converted to logMAR for analysis, a slit-lamp and dilated fundus examination, and optical coherence tomography (OCT) scans using an Avanti RTVue XR system (OptoVue, Fremont, CA, USA) before brolucizumab treatment (baseline) and at each follow-up visit [16, 17]. Crossline scan protocol centered on the fovea was used. The presence of SRF or IRF was recorded. CRT was defined as the average thickness of the central 1-mm diameter area of the fovea, which was automatically measured by the OCT device. The height of pigment epithelial detachment (PED) was defined as the longest vertical distance from Bruch’s membrane to the RPE border in a horizontal or vertical cross line scan centered on the fovea [15]. PED height was measured manually using the caliper tool available on the OCT device. It was measured twice by a retinal specialist (C.C.W.), and the average of the two measurements was used. Treatment regimen and follow-up frequency were based on the discretion of each doctor. The patients were instructed on the signs and symptoms of IOI, and were advised to visit the clinic for monitoring of adverse events one week after each brolucizumab injection and whenever any events occurred, as well as monthly evaluations to assess efficacy. Retreatment criteria varied among physicians. Some required a loading dose of three injections, while others based on presence of SRF or IRF on OCT with/without vision decline. For the latter group, if there was complete resolution of fluid after the first brolucizumab injection, then the patient would not receive another injection until there was a recurrence of fluid.

The primary outcomes were changes in mean BCVA and CRT from baseline to the third month. The secondary outcomes included the incidence of IOI, proportions of patients with SRF and IRF, and change in PED height from baseline to the third month. In addition, the proportions of patients achieving at least 0.1, 0.2, and 0.3 logMAR (equivalent to 5, 10, 15 ETDRS letters) visual gain during the first 3 months were also analyzed [17].

Data were analyzed using SPSS (version 20.0; SPSS, Inc., Chicago, IL, USA). Continuous variables were described as means and standard deviations (SDs). Paired data in comparison with baseline were analyzed using the paired t-test. Categorical variables were described as absolute values and percentages. McNemar’s test was used to compare differences in SRF and IRF rates before and after treatment. The significance level was considered as p < .05 in all tests.

## Results

**Fig 1:**
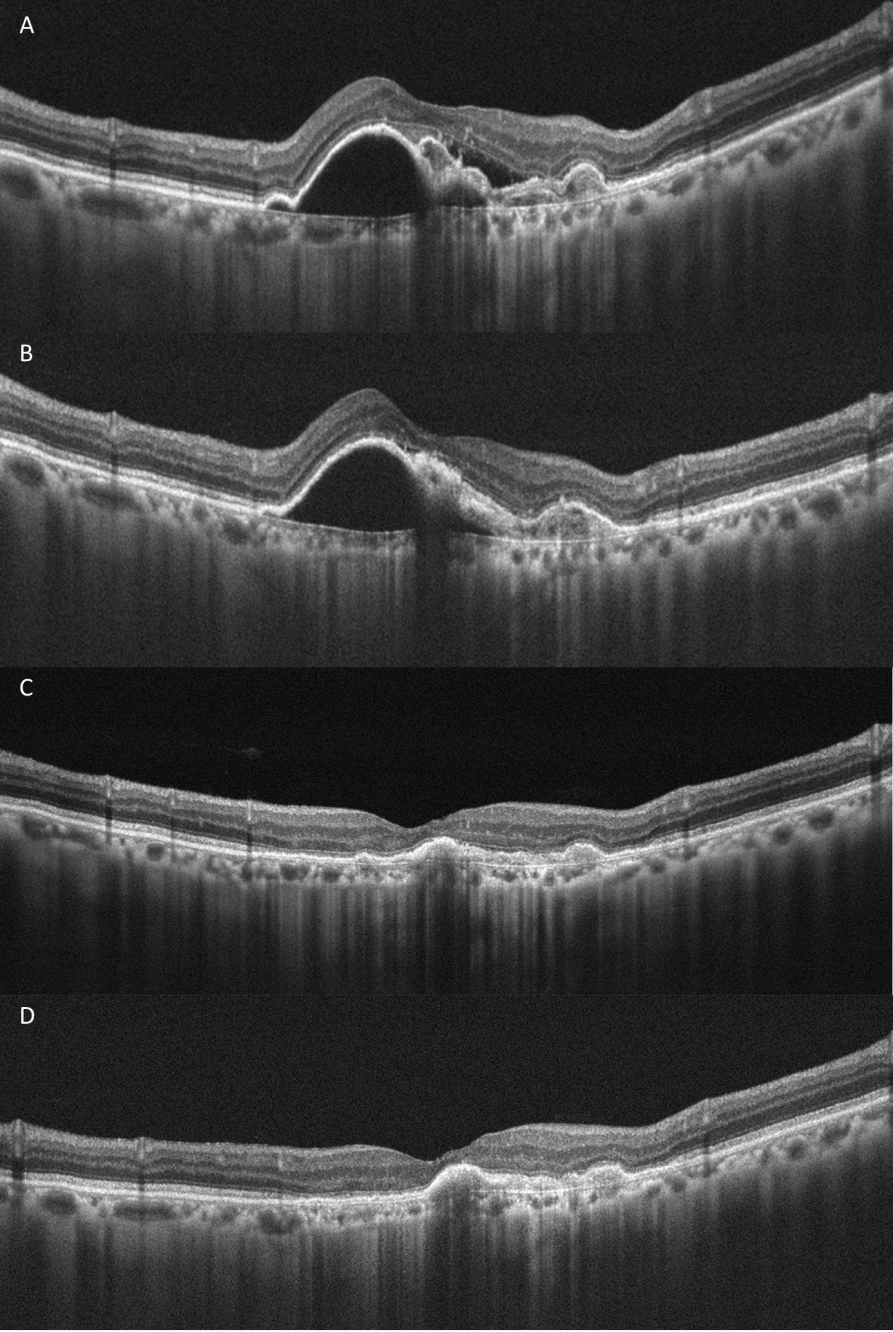
A representative PCV case of 83 years old man with a 95-month treatment period and 27-month persistent fluid. Persistent subretinal and intraretinal fluid and peaked PED before switching to brolucizumab were shown (A). He had 3 monthly brolucizumab injections. Resolution of the subretinal and intraretinal fluid and mildly decreased PED height were demonstrated one month after switching to brolucizumab (B). Persistent absence of the subretinal and intraretinal fluid and significant decreased PED height were demonstrated two (C) and three (D) months after switching to brolucizumab.

A total of 38 patients (40 eyes) with a mean (±SD) age of 76.3 (±10.84) years who were followed for 3 months after switching to brolucizumab were included. Of the 38 patients, 27 were males. Among the 40 study eyes, 15 were phakic, and the remaining 25 were pseudophakic. The percentages of nAMD and PCV among the 40 eyes were 60% and 40%, respectively. Eight eyes among the patients with PCV had undergone photodynamic therapy. The mean periods (±SD) of treatment and persistent fluid before switching to brolucizumab were 55.9 (±30.39, range 4 to 173) and 20.5 (±16.56, range 2 to 65) months, respectively. The number (mean±SD) of previous other anti-VEGF injections were 21.4±13.13, with specific breakdowns as follows: aflibercept injections were 17.2±10.88, ranibizumab injections were 3.5±4.58, and bevacizumab injections were 0.7±1.58. There were mean (±SD) 2.2±0.92 brolucizumab injections per eye, of which 11 eyes had only one injection during the first 3 months. A representative case is shown in Fig 1.

**Fig 2:**
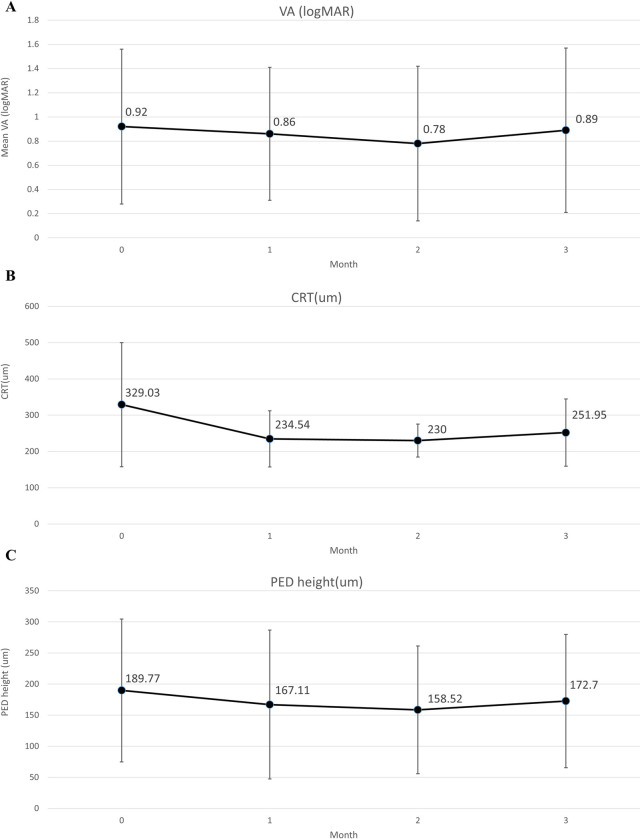
(A) Change in VA by month, (B) Change in CRT by month, (C) Change in PED height by month. Abbreviations: CRT, central retinal thickness; PED, retinal pigment epithelium detachment; VA, visual acuity.

**Fig 3:**
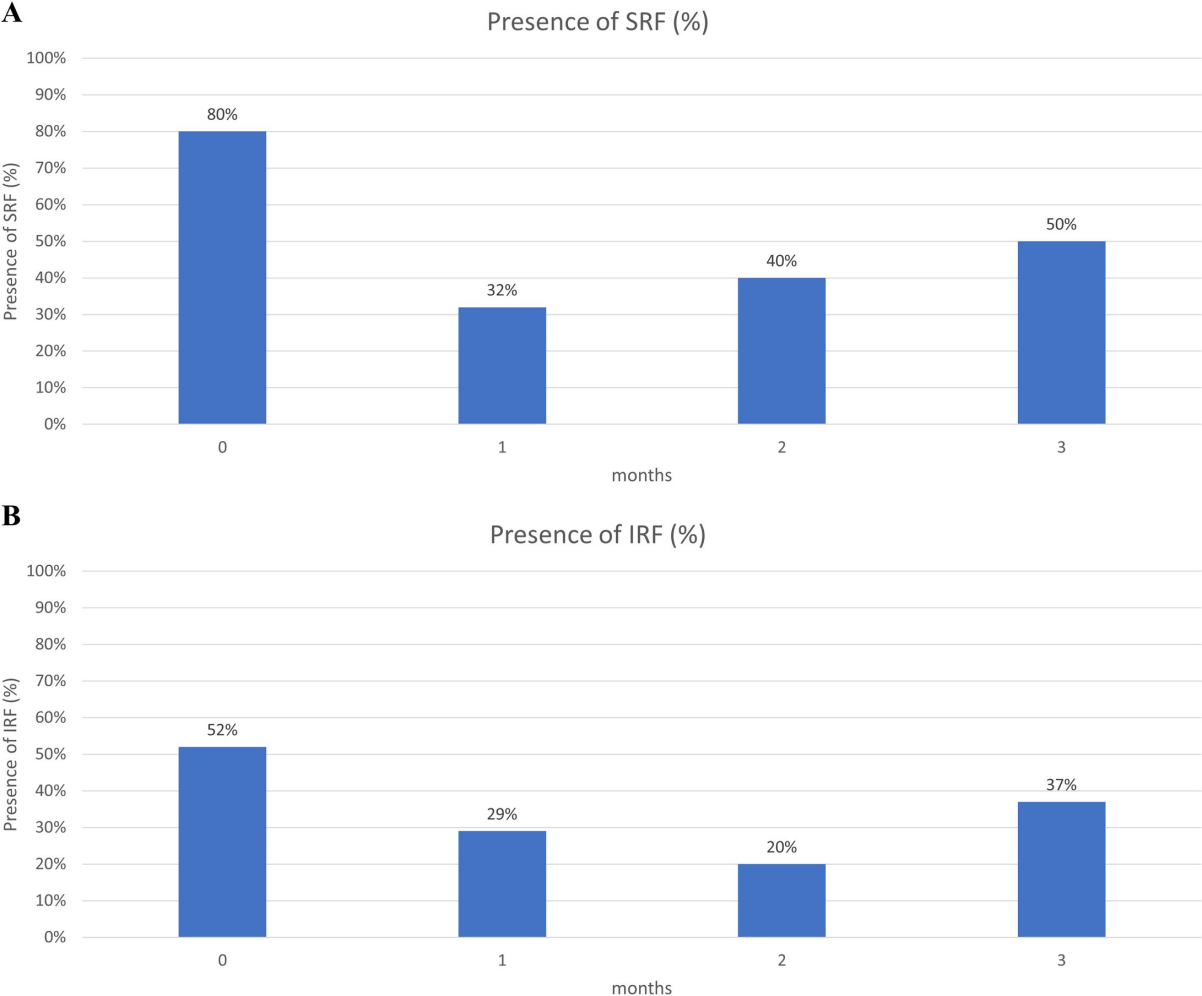
(A) Change in SRF by month, (B) Change in IRF by month. Abbreviations: IRF, intraretinal fluid; SRF, subretinal fluid.

The patients had a significant reduction in CRT and resolution of IRF and SRF from baseline to the third month. There were numerical improvements in mean BCVA and PED height, but they were not significant. At baseline, 32 and 21 eyes had SRF and IRF, respectively. At the third month, 12 (37.5%) and 6 (28.5%) eyes had resolution of SRF and IRF, respectively (Table 1). Changes in VA, CRT, PED, IRF, and SRF by month are shown in Figs 2 and 3 and summarized in S1 Table. There were significant reductions in CRT and resolution of SRF in each month, and in IRF starting in the second month. There were improvements in VA and PED in each month, but they were not significant. Subgroup analysis of nAMD and PCV was performed, which showed a significant reduction in CRT but no significant improvement in mean BCVA or PED height in either group. In addition, among the 40 study eyes, two were vitrectomized. After excluding these two vitrectomized eyes, the main outcomes still showed the same trend, as illustrated in S2 Table. Similarly, the main outcomes exhibited a consistent trend when we selected the right eye from patients who had both eyes switching to brolucizumab and reanalyzed these 38 eyes, as illustrated in S3 Table. We also performed subgroup analyzes for eyes receiving 1, 2, and at least 3 injections, and the results remained consistent, as illustrated in S4 Table.

**Table 1:** Change of functional and anatomical parameters after brolucizumab injections at 3 months.

N = 40		Baseline	3rd Month	*P* value
**VA (logMAR)**	Mean(SD)	0.92(0.64)	0.89(0.68)	.608
**CRT (um)**	Mean(SD)	329.0(171.18)	251.2(92.76)	.006
**PED height (um)**	Mean(SD)	189.8(114.94)	172.7(107.13)	.173
**SRF**				
Present	n(%)	32(80%)	20(50%)	< .001
Absent	n(%)	8(20%)	20(50%)	
**IRF**				
Present	n(%)	21(52.5%)	15(39.5%)	.031
Absent	n(%)	19(47.5%)	23(60.5%)	

**Abbreviations**: CRT, central retinal thickness; IRF, intraretinal fluid; PED, retinal pigment epithelium detachment; SRF, subretinal fluid; VA, visual acuity.

**Fig 4:**
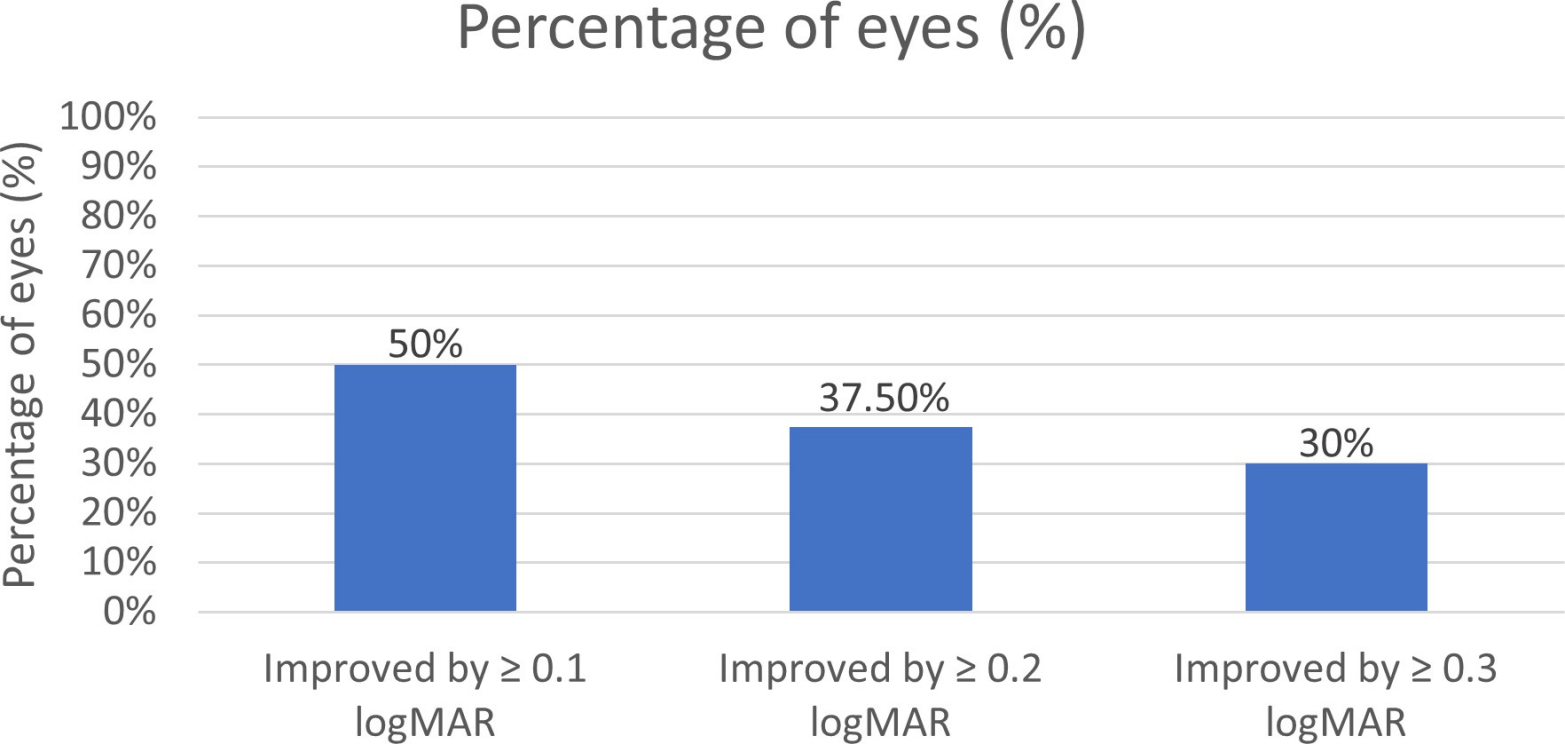
Percentage of the 40 eyes with BCVA gains during the first 3 months after brolucizumab treatments. Abbreviations: BCVA, best-corrected visual acuity.

The percentages of achieving at least 0.1, 0.2, and 0.3 logMAR (equivalent to 5, 10, 15 ETDRS letters) visual gain were 50%, 37.5%, and 30%, respectively, during the first 3 months of follow-up, as shown in Fig 4. In addition, 75% of the eyes achieved resolution of both IRF and SRF during the 3-month follow-up period. No IOI occurred in these patients.

## Discussion

This study provides our real-world experience of the short-term efficacy and safety outcomes of recalcitrant nAMD patients who received brolucizumab treatment in Taiwan. Although visual gain was not significant after switching to brolucizumab, half of the patients still gained at least 0.1 logMAR BCVA during the first 3 months of follow-up. In addition, improvements in anatomical outcomes, including CRT, SRF, and IRF were significant in the third month. Moreover, none of the patients experienced IOI events.

Most real-world studies have not shown a significant visual gain after switching to brolucizumab in refractory nAMD patients, which is consistent with our findings [10–12, 14, 15, 18]. On the other hand, the BRAILLE study, with a mean follow-up period of 7.3 ± 2.2 weeks and 1+ Pro re nata (PRN) regimen, did demonstrate significant visual improvements [13]. Of note, the mean number of previous other anti-VEGF injections was lower in the BRAILLE study (8.63 ± 4.74) than in the other real-world studies, including the present study (21.4±13.13). Compared to the BRAILLE study, the lower visual gain in the present study may be related to a longer treatment history (55.9±30.39 months) and persistent fluid (20.5±16.56 months). In addition, there was a trend toward lesser anatomical and functional improvements in the third month compared to the first and second months in the current study. This may be related to the recurrence of disease activity in the patients who only had one brolucizumab injection.

Both the HAWK and HARRIER trials demonstrated a significant reduction in CRT and fluid resolution in nAMD patients receiving brolucizumab treatment compared to those receiving aflibercept treatment [7, 8]. Real-world data, including the present study, have also confirmed that brolucizumab was more potent in drying the macula than other anti-VEGF agents [10–15, 18]. These data are consistent with our findings that brolucizumab could still reduce SRF and IRF in recalcitrant nAMD patients who had nearly 2 years of persistent fluid. In addition, Boltz et al. and Rispoli et al. reported significant reductions in PED height after switching to brolucizumab in refractory nAMD patients [15, 18]. Our data also showed a trend of a reduction in PED, although without reaching significance.

Real-world studies focusing on PCV refractory to previous anti-VEGF treatment showed significant fluid reduction but non-significant improvement in BCVA after switching to brolucizumab, which is consistent with our findings [19, 20]. The regression rate of polypoidal lesions was reported up to 78.9% after three monthly injections of brolucizumab in treatment-naïve PCV and 26.7% at 18 months in recalcitrant PCV [20, 21]. However, in the present study, it is not possible to evaluate the effect on polyp regression as ICGA was not performed after switching to brolucizumab. Yet, 62.5% of PCV eyes had reduction of PED height at the third month after switching to brolucizumab which might indicate a favorable response of polyps.

A post-hoc review of the HAWK and HARRIER trials reported an incidence rate of IOI of 4.6% with brolucizumab compared to 1.1% with aflibercept [22]. In addition, the median time to onset was 25.5 days (range: 1–91 days) from the last brolucizumab injection. Possible risk factors for IOI included female sex, Japanese ethnicity, and a history of IOI and/or retinal vascular occlusion within the 12 months before the initiation of brolucizumab [9]. Among real-world studies, the highest incidence of IOI has been reported in Japanese patients, followed by Caucasian and Indian patients [10–15]. In order to detect IOI events early, we instructed patients on the signs and symptoms of IOI and advised them to visit the clinic for adverse event monitoring one week after each brolucizumab injection and whenever any events occurred, as well as monthly evaluations to assess efficacy. A comprehensive ophthalmic examination, including slit-lamp examination and funduscopy after pupil dilation was performed to detect any signs of IOI at each visit. Therefore, the chance of missing cases with IOI in this study cohort is low. Although no IOI events occurred in this study, the results should be interpreted with caution due to the small sample size, male predominance, and short follow-up period, which may not be representative of the Chinese IOI rate or allow for cross-racial comparisons.

This study has several limitations. The retrospective nature and varied treatment approaches of each doctor may have affected the outcomes. In addition, the small sample size, few brolucizumab injections per eye and short review period limit the conclusiveness of our findings, and may not be sufficient to identify the relatively rare occurrence of IOI. Furthermore, the manually measured PED height may have the hazards of bias in itself. Despite these limitations, our results represent the real-world data regarding the short-term efficacy and safety of brolucizumab treatment in Taiwanese patients with recalcitrant nAMD.

## Conclusion

The findings of this Taiwan real-world study suggest that brolucizumab was effective in treating recalcitrant nAMD patients with a long duration of persistent fluid (20.5±16.56 months), by demonstrating beneficial effects on morphological outcomes and that half of the patients gained at least 0.1 logMAR BCVA during the first 3 months of follow-up. Even though none of the patients had IOI in this study, further studies with a larger sample size and longer follow-up period are needed to clarify the safety profile and efficacy of brolucizumab.

## Supporting information

S1 File Raw data for replicating this study findings. (XLSX)

S1 Table Change in functional and anatomical parameters by month. Abbreviations: CRT, central retinal thickness; IRF, intraretinal fluid; PED, retinal pigment epithelium detachment; SRF, subretinal fluid; VA, visual acuity. *Paired T Test. **Compare to the baseline, McNamer test.
(DOCX)

S2 Table Change of functional and anatomical parameters after brolucizumab injections at 3 months (excluding vitrectomized eyes). Abbreviations: CRT, central retinal thickness; IRF, intraretinal fluid; PED, retinal pigment epithelium detachment; SRF, subretinal fluid; VA, visual acuity.
(DOCX)

S3 Table Change of functional and anatomical parameters after brolucizumab injections at 3 months (selecting the right eye from patients who had both eyes switching to brolucizumab). Abbreviations: CRT, central retinal thickness; IRF, intraretinal fluid; PED, retinal pigment epithelium detachment; SRF, subretinal fluid; VA, visual acuity.
(DOCX)

S4 Table Change of functional and anatomical parameters after brolucizumab injections at 3 months (subgroup analyzes for eyes receiving 1, 2, and at least 3 brolucizumab injections). Abbreviations: CRT, central retinal thickness; PED, retinal pigment epithelium detachment; VA, visual acuity.
(DOCX)

## Abbreviations:

PCV: polypoidal choroidal vasculopathy
PED: retinal pigment epithelium
