# Supplementary material for: Brolucizumab in recalcitrant neovascular age-related macular degeneration–real-world data in Chinese population
Source: PLoS One. 2024 Apr 2;19(4):e0301096. doi: 10.1371/journal.pone.0301096 (PMC10986944; doi:10.1371/journal.pone.0301096)
Supplement: S1 Table — Abbreviations: CRT, central retinal thickness; IRF, intraretinal fluid; PED, retinal pigment epithelium detachment; SRF, subretinal fluid; VA, visual acuity. *Paired T Test. **Compare to the baseline, McNamer test. (DOCX) [file pone.0301096.s002.docx]

| VA change | | | | |
| --- | --- | --- | --- | --- |
| Month | Difference | SD | N | *P* value* |
| 1 | -0.10 | 0.31 | 28 | .114 |
| 2 | -0.07 | 0.31 | 25 | .251 |
| 3 | -0.03 | 0.31 | 40 | .608 |
| CRT change | | | | |
| 1 | -114.61 | 148.41 | 28 | <.001 |
| 2 | -125.92 | 195.04 | 25 | .004 |
| 3 | -77.08 | 167.98 | 40 | .006 |
| Height of PED change | | | | |
| 1 | -14.66 | 49.30 | 28 | .127 |
| 2 | -31.04 | 75.87 | 25 | .052 |
| 3 | -17.07 | 77.75 | 40 | .173 |
| Presence of SRF | | | | |
| month | Percentage |  | N | *P* value** |
| 1 | 0.32 |  | 28 | <.001 |
| 2 | 0.4 |  | 25 | <.001 |
| 3 | 0.5 |  | 40 | <.001 |
| Presence of IRF | | | | |
| 1 | 0.29 |  | 28 | .063 |
| 2 | 0.2 |  | 25 | .039 |
| 3 | 0.38 |  | 40 | .031 |

**S1 Table.** Change in functional and anatomical parameters by month.

**Abbreviations:** CRT, central retinal thickness; IRF, intraretinal fluid; PED, retinal pigment epithelium detachment; SRF, subretinal fluid; VA, visual acuity.

*Paired T Test

**Compare to the baseline, McNamer test
