# Supplementary material for: Brolucizumab in recalcitrant neovascular age-related macular degeneration–real-world data in Chinese population
Source: PLoS One. 2024 Apr 2;19(4):e0301096. doi: 10.1371/journal.pone.0301096 (PMC10986944; doi:10.1371/journal.pone.0301096)
Supplement: S2 Table — Abbreviations: CRT, central retinal thickness; IRF, intraretinal fluid; PED, retinal pigment epithelium detachment; SRF, subretinal fluid; VA, visual acuity. (DOCX) [file pone.0301096.s003.docx]

**S2 Table.** Change of functional and anatomical parameters after brolucizumab injections at 3 months (excluding vitrectomized eyes).

| **N = 38** |  | **Baseline** | **3rd Month** | ***P* value** |
| --- | --- | --- | --- | --- |
| **VA (logMAR)** | Mean(SD) | 0.94(0.64) | 0.92(0.68) | .666 |
| **CRT (um)** | Mean(SD) | 328.0(174.54) | 251.5(94.15) | .010 |
| **PED height (um)** | Mean(SD) | 193.7(116.59) | 176.4(108.71) | .189 |
| **SRF** |  |  |  |  |
| Present | n(%) | 30(78.9%) | 19(50%) | .001 |
| Absent | n(%) | 8(21.1%) | 19(50%) |  |
| **IRF** |  |  |  |  |
| Present | n(%) | 20(52.6%) | 15(39.5%) | .063 |
| Absent | n(%) | 18(47.4%) | 23(60.5%) |  |

**Abbreviations:** CRT, central retinal thickness; IRF, intraretinal fluid; PED, retinal pigment epithelium detachment; SRF, subretinal fluid; VA, visual acuity.
