# Supplementary material for: Brolucizumab in recalcitrant neovascular age-related macular degeneration–real-world data in Chinese population
Source: PLoS One. 2024 Apr 2;19(4):e0301096. doi: 10.1371/journal.pone.0301096 (PMC10986944; doi:10.1371/journal.pone.0301096)
Supplement: S3 Table — Abbreviations: CRT, central retinal thickness; IRF, intraretinal fluid; PED, retinal pigment epithelium detachment; SRF, subretinal fluid; VA, visual acuity. (DOCX) [file pone.0301096.s004.docx]

**S3 Table.** Change of functional and anatomical parameters after brolucizumab injections at 3 months (selecting the right eye from patients who had both eyes switching to brolucizumab).

| **N = 38** |  | **Baseline** | **3rd Month** | ***P* value** |
| --- | --- | --- | --- | --- |
| **VA (logMAR)** | Mean(SD) | 0.95(0.64) | 0.92(0.68) | .593 |
| **CRT (um)** | Mean(SD) | 334.0(174.25) | 254.6(94.35) | .007 |
| **PED height (um)** | Mean(SD) | 194.0(116.25) | 175.1(109.09) | .152 |
| **SRF** |  |  |  |  |
| Present | n(%) | 30(78.9%) | 19(50%) | .001 |
| Absent | n(%) | 8(21.1%) | 19(50%) |  |
| **IRF** |  |  |  |  |
| Present | n(%) | 21(55.3%) | 15(39.5%) | .031 |
| Absent | n(%) | 17(44.7%) | 23(60.5%) |  |

**Abbreviations:** CRT, central retinal thickness; IRF, intraretinal fluid; PED, retinal pigment epithelium detachment; SRF, subretinal fluid; VA, visual acuity.
