# Supplementary material for: Brolucizumab in recalcitrant neovascular age-related macular degeneration–real-world data in Chinese population
Source: PLoS One. 2024 Apr 2;19(4):e0301096. doi: 10.1371/journal.pone.0301096 (PMC10986944; doi:10.1371/journal.pone.0301096)
Supplement: S4 Table — Abbreviations: CRT, central retinal thickness; PED, retinal pigment epithelium detachment; VA, visual acuity. (DOCX) [file pone.0301096.s005.docx]

**S4 Table.** Change of functional and anatomical parameters after brolucizumab injections at 3 months (subgroup analyzes for eyes receiving 1, 2, and at least 3 brolucizumab injections).

| **Group 1 (receiving 1 injection)** | | | | |
| --- | --- | --- | --- | --- |
| **N = 11** |  | **Baseline** | **3rd Month** | ***P* value** |
| **VA (logMAR)** | Mean(SD) | 1.13(0.72) | 1.20(0.75) | .434 |
| **CRT (um)** | Mean(SD) | 285.8(69.95) | 231.4(81.35) | .082 |
| **PED height (um)** | Mean(SD) | 147.3(87.75) | 155.7(97.37) | .605 |
| **Group 2 (receiving 2 injections)** | | | | |
| **N = 15** | | | | |
| **VA (logMAR)** | Mean(SD) | 0.87(0.71) | 0.83(0.77) | .664 |
| **CRT (um)** | Mean(SD) | 345.8(227.13) | 263.1(97.77) | .193 |
| **PED height (um)** | Mean(SD) | 205.7(126.87) | 194.9(132.68) | .594 |
| **Group 3 (receiving at least 3 injections)** | | | | |
| **N = 14** |  |  |  |  |
| **VA (logMAR)** | Mean(SD) | 0.80(0.47) | 0.72(0.43) | .235 |
| **CRT (um)** | Mean(SD) | 345.0(162.52) | 256.2(99.52) | .028 |
| **PED height (um)** | Mean(SD) | 206.1(119.45) | 162.3(85.18) | .096 |

**Abbreviations**: CRT, central retinal thickness; PED, retinal pigment epithelium detachment; VA, visual acuity.
